# An evaluation of lower urinary tract symptoms in diabetic patients: a cross-sectional study

**DOI:** 10.1186/s12894-022-01133-1

**Published:** 2022-11-10

**Authors:** Hala Qasrawi, Mahmoud Tabouni, Sara W. Almansour, Mohammad Ghannam, Amjad Abdalhaq, Faris Abushamma, Amer A. Koni, Sa’ed H. Zyoud

**Affiliations:** 1 Department of Radiology, An-Najah National University Hospital, Nablus, 44839 Palestine; 2 Department of Anaesthesia, An-Najah National University Hospital, Nablus, 44839 Palestine; 3 Arab Women Union Hospital, Nablus, 44839 Palestine; 4 UNRWA, Jerusalem, Palestine; 5 Nablus Speciality Hospital, Nablus, 44839 Palestine; 6 Department of Medicine, College of Medicine and Health Sciences, An-Najah National University, Nablus, 44839 Palestine; 7 Department of Urology, An-Najah National University Hospital, Nablus, 44839 Palestine; 8 Division of Clinical Pharmacy, Hematology and Oncology Department, An-Najah National University Hospital, Nablus, 44839 Palestine; 9 Department of Clinical and Community Pharmacy, College of Medicine and Health Sciences, An-Najah National University, Nablus, 44839 Palestine; 10 Clinical Research Center, An-Najah National University Hospital, Nablus, 44839 Palestine; 11 Poison Control and Drug Information Center (PCDIC), College of Medicine and Health Sciences, An-Najah National University, Nablus, 44839 Palestine

**Keywords:** LUTS, Prevalence, Diabetes mellitus, UDI-6, IIQ-7, Urinary distress symptoms

## Abstract

**Background:**

Lower urinary tract symptoms (LUTS) are common among diabetic patients and represent hidden and mysterious morbidity. The pathophysiology of LUTS among diabetes mellitus (DM) patients is multifactorial. Importantly, LUTS is known to cause physical and psychological distress. Thus, this study describes LUTS among DM patients, investigates factors that may associate with it, and assesses the possible relationship between LUTS and the quality of life of diabetics.

**Methods:**

Over 6 months, data were collected from 378 diabetic patients in primary health care clinics. Demographic and clinical characteristics, Urogenital Distress Inventory-6 (UDI-6), and Incontinence Impact Questionnaire-7 (IIQ-7) were used to collect data. Univariate and multivariate analyses were performed.

**Results:**

Three hundred seventy-eight participants were included in this study. (29.9%) were (58–67) years old. 49% were female. Half of the cohort was overweight, and a third were obese. 81% were Type 2 DM. Almost all of them are on medical treatment. A median score of 5.50 (2.00–8.00) for the UDI-6 scale and a median score of 5 (0.00–10.00) for the IIQ-7 scale were reported. Multiple linear regression models showed that residency (*p* = 0.038) and regular exercise (*p* = 0.001) were significantly and negatively correlated with the UDI-6 score, while female gender (*p* = 0.042), insulin use (*p* = 0.009) and the presence of comorbidities (*p* = 0.007) were positively correlated with this score. Furthermore, age (*p* = 0.040) and body mass index (BMI) (*p* < 0.001) were significantly and positively associated with the IIQ-7 score.

**Conclusion:**

LUTS is significant morbidity among DM patients. Factors such as age, BMI, and co-morbidities exacerbate LUTS, which can be modified and controlled. On the other hand, regular exercise and weight loss strategies help diabetic patients to improve LUTS.

## Background

Diabetes mellitus (DM) is a common health problem in developing countries [1]. Type 1 and type 2 DM have several manifestations that affect vital organs such as the kidney, heart, and vascular systems [2]. Thus, DM may lead to significant morbidity and mortality attributed to heart attacks, end-stage renal failure, and peripheral vascular disease. Nevertheless, DM is known to affect the urinary tract by several mechanisms, such as recurrent urinary tract infections (UTIs), urinary tract calculi, and bladder dysfunction [3, 4]. Recently, several studies linked DM to Lower urinary tract symptoms (LUTS), investigating factors that may exacerbate LUTS in DM patients, such as gender, the level of Hemoglobin A1C (Hba1c), and body mass index (BMI) [5, 6]. Moreover, LUTS has been proven to negatively affect the quality of life (QoL) among different cohorts of patients [7–9]. Therefore, the prevalence of LUTS among DM and factors that may exacerbate LUTS are important concerns that need to be explored and studied.

LUTS is a broad spectrum of symptoms, including storage, voiding, and incontinence [10]. In addition, LUTS in diabetic patients may represent underlying pathology such as UTIs [11–13]. Nevertheless, LUTS may represent a denovo phenomenon reflecting bladder dysfunction or bladder outlet problem, especially in men with concomitant prostate problems [14–16]. Thus, LUTS in DM requires detailed history taking and physical examination before proceeding with invasive procedures. Furthermore, lifestyle modification and risk adjustment strategies may help relieve LUTS. For instance, weight loss, decreased caffeinated drink intake, and decreased fluid intake are all strategies that have been proven to alleviate LUTS [17–19]. However, particularly in DM, further studies are required to show factors that may cause or exacerbate LUTS to create a DM-focused strategy to avoid LUTS.

Generally, DM bothers patients when complications appear [20–23]. LUTS also is known to cause physical and psychological distress [9, 24]. Thus, any measures to improve the DM patients’ LUTS symptoms are paramount. However, tight control of DM is not known if it directly helps with LUTS [25–27]. Regular exercise, weight loss, and fluid adjustment were found to improve LUTS but not specifically in DM patients. Thus, a detailed analysis of LUTS in DM is required to uncover modifiable risk factors that may contribute to LUTS, thus preventing minor but significant morbidity.

In Palestine, no studies show the prevalence of LUTS among DM patients nor explore risk factors that may cause LUTS. Thus, this study aims to explore the LUTS problem among DM patients attending primary health care centers given the prevalence of LUTS and risk factors. The current study assesses the possible impact of LUTS and the quality of life among diabetic patients by analyzing the association between the severity of LUTS measured by the UDI-6 and the QoL of the patients measured by IIQ-7. We aim that the results of this study may help to help DM patients by identifying adjustable risk factors for LUTS and, consequently, alleviating their symptoms. Furthermore, in Palestine, the results of this study may act as a trigger point for policymakers and stakeholders, so a strategy to screen and identify LUTS early may be put in place, which helps DM patients to recognize their symptoms earlier and get appropriate counseling and treatment.

## Methods

### Study design

A cross-sectional study was designed to assess LUTS prevalence among diabetic patients and its risk factors. A questionnaire-based interview was used to collect data from study participants.

### Study setting and study population

The survey was carried out in two diabetic clinics in primary health care centers of the Ministry of Health, Nablus, Palestine. The target population was diabetic patients. Data were collected between May 2021 and October 2021. During interviews, we committed to all infection control measures due to the COVID-19 pandemic including social distancing and masks. The appointments were made between 8 AM and 3 PM, which are the official hours assigned to conducting follow-up appointments for diabetic patients in the clinics studied.

### Sample size and sampling method

During the study period, the approximate number of patients who visited the diabetes clinics at the Nablus health center was 2000. This number was used to determine the sample size for the current analysis. Using the Raosoft sample size calculator, a sample size of 323 was determined by setting the response distribution at 0.50, the error margin at 5% and the confidence interval at 95%. The target sample size was increased to 378 participants to improve the reliability of the research and decrease erroneous outcomes. The convenience sampling technique was used to achieve the target sample.

### Inclusion and exclusion criteria

All patients who were confirmed to have DM by laboratory tests were included. These patients are also required to visit the diabetes clinics in Nablus medical center due to DM or its complications. Patients with an established diagnosis of a urogenital condition, a history of urological surgery or recurrent UTIs, or those with a psychiatric disease were excluded.

### Data collection tool

The data collection tool was an Arabic-language questionnaire composed of three sections. The first section was about the demographic features. The structured questionnaire in this study was used in previously published studies [28–33]. Indeed, we asked about age (< 38, 38–47 years, 48–57 years, 58–67 years, or ≥ 68 years [34]), marital status, weight, height, residency, smoking status, alcohol intake, employment status, level of education, income, physical exercise, presence of any medical disorders, long-term medication use, type of treatment, duration of DM and last HbA1c reading. BMI was measured as weight in kilograms (kg) divided by height in meters squared (m2), based on self-reported weight and height. Based on their calculated (BMI), participants were classified into four groups: obese (BMI ≥ 30 kg/m^2^), overweight (BMI = 25–29.9 kg/m^2^), normal weight (BMI = 18.5–24.9 kg/m^2^), or underweight (BMI < 18.5 kg/m^2^) [35].

In the short form of the second section, the Urogenital Distress Inventory 6 (UDI-6) short form, which is similar to its full version, assesses the severity of urinary distress symptoms based on the level of discomfort during the past month [28–30]. UDI-6 contains six multiple-choice questions that cover three areas: irritative symptoms (questions 1–2), stress symptoms (questions 3–4), and obstructive or discomfort symptoms (questions 5–6). The participants responded to each section using a scale of four options: ‘greatly’, ‘moderately’, ‘a little bit’, and ‘not at all’. Each answer ranged from zero to three points, with ‘greatly’ receiving three points and ‘not at all’ receiving zero points. Therefore, the highest possible UDI score is 18. The internal consistency of UDI was 0.720, tested by the Cronbach alpha coefficient. We obtained permission from the developer to use the Arabic version of this tool in our study [29].

The third section included the short versions of the Incontinence Impact Questionnaire-7 (IIQ-7). IIQ-7 is a tool designed to assess the impact of urinary incontinence on QoL. Similarly, to its full version, IIQ-7 focuses on four areas: physical activity (questions 1–2), travel (questions 3–4), social relations (question 5), and emotional well-being (questions 6–7). The severity of symptoms is rated on a scale from zero to three, while zero is the least severe, and three is the most severe [30]. The highest possible IIQ-7 score is 21. The Cronbach alpha coefficient previously tested this tool; its internal consistency was 0.894. The developer obtained approval to include the Arabic version of this tool in our study.

All scores for UDI-6 and IIQ-7 were converted to a scale of 0 to 100 to compare measures with each other [36]. UDI-6 and IIQ-7 are valid and reliable questionnaires to evaluate subjective phases of urinary incontinence and the impact of LUTS on QoL. Both tests are feasible and have a level of validation according to the ICI grades. The Arabic version of this scale was validated and used in previous studies [28–30]. They helped determine the severity of incontinence, the efficiency of treatment, and make a management plan [37, 38].

### Ethics

Approvals from the Institutional Review Board of An-Najah National University and the Ministry of Health were obtained to carry out the current investigation. Before the interviews, all study aspects were discussed in detail with all patients, and we confirmed that their confidentiality was secured. After that, verbal consent was obtained.

### Statistical analysis

This study used the Statistical Package for the Social Sciences (IBM-SPSS) version 21 for data analysis. We presented the characteristics as percentages and frequencies, and the questionnaire scores as medians and interquartile ranges. We used the Kolmogorov–Smirnov test to establish the normality of the variables. The Mann–Whitney and Kruskal–Wallis tests were also used to test for differences in the scores between different categories of participants. The correlations between the different scales were evaluated by Spearman correlation. Furthermore, multiple linear regressions were performed to predict the variables that had a significant relationship with UDI-6 and IIQ-7 scores. The P-value of < 0.05 was assumed significant.

## Results

### Demographic

A total of 378 participants participated in the study. The highest number of subjects was 58–67 years (29.9%) and had an overweight BMI (49.5%). In addition, 50.8% of the participants were men and more than half were smokers (57.7%). The majority of them were married, and approximately a third had high school educational levels (61.6%, and 31.5%, respectively), with a 46.0% living in the village area (Table 1).

**Table 1:** Relationship between the participants’ characteristics and their UDI score and IIQ-7 score

Characteristic	Frequency (%) N = 378	UDI-6 score Median [Q1–Q3]	Mean Rank (UDI-6 score)	P-value*	IIQ-7 score Median [Q1–Q3]	Mean Rank (IIQ-7 score)	P-value*
*Age category*				**0.003**^**a**^			** < 0.001**^**a**^
Under 38	62 (16.4)	3 (2–6)	150.07		0 (0–3)	130.19	
38–47	34 (9.0)	5 (4–6.3)	185.13		3 (0–4.5)	146.24	
48–57	93 (24.6)	6 (1.5–8)	185.97		4 (0–9)	183.01	
58–67	113 (29.9)	6 (2–9)	191.62		7 (0–10.5)	202.59	
68 and older	76 (20.1)	6 (3–11)	224.78		9 (5–11)	245.72	
*Sex*				**0.007**^**b**^			** < 0.001**^**b**^
Male	192 (50.8)	5 (2–7)	174.69		3 (0–8)	169.36	
Female	186 (49.2)	6 (2–10)	204.79		7 (0–11)	210.29	
*Smoking Status*				0.91^**b**^			0.072 ^b^
Smoker	218 (57.7)	6 (2–80	188.76		4 (0–8)	177.88	
Non-smoker	160 (42.3)	5 (2–9)	190.04		7 (0–10.5)	198.03	
*BMI*				**0.010**^**a**^			** < 0.001**^**a**^
Normal	74 (19.6)	4 (2–6)	158.34		0 (0–6.3)	138.99	
Overweight	187 (49.5)	6 (2–8)	190.78		4 (0–9)	180.52	
Obese	117 (31.0)	6 (2–10)	207.17		8 (3–12)	235.80	
*Marital Status*				**0.008**^**b**^			**0.022**^**b**^
Single	145 (38.4)	6.0 (3.0–11.0)	208.27		7.0 (0.0–11.0)	205.55	
Married	233 (61.6)	5.0 (2.0–7.5)	177.82		4.0 (0.0–9.0)	179.51	
*Residency*				**0.037**^**a**^			**0.027**^**a**^
City	168 (44.4)	4 (2–7)	174.47		3 (0–8)	172.92	
Village	174 (46.0)	6 (2–9.25)	198.43		6 (0–10)	202.68	
Refugee camp	36 (9.5)	6 (3–12)	216.49		7 (0–9)	203.19	
*Educational level*				**0.043**^**b**^			** < 0.001**^**b**^
School	219 (57.9)	6.0 (2.0–10.0)	199.15		7.0 (1.0–11.0)	212.36	
University	159 (42.1)	5.0 (2.0–7.0)	176.20		2.0 (0.0–8.0)	158.01	
*Job*				**0.013**^**b**^			** < 0.001**^**b**^
Unemployed	248 (65.6)	6.0 (2.0–9.8)	199.51		7.0 (0.0–11.0)	207.5	
Employed	130 (34.4)	4.0 (2.0–7.0)	170.41		2.0 (0.0–7.0)	155.16	
*Income*				**0.017**^**a**^			** < 0.001**^**a**^
Less than 2000 NIS	171 (45.2)	5 (2–8)	185.84		7 (0–11)	210.67	
2000–5000 NIS	139 (36.8)	6 (3–9)	208.70		5.5 (0–9)	188.24	
5000–10,000 NIS	58 (15.3)	4 (3–6.25)	163.16		1 (0–4.25)	142.58	
More than 10,000	10 (2.6)	3 (0–9)	137.90		0.5 (0–4)	117.20	
*Type of insurance*				** < 0.001**^**a**^			** < 0.001**^**a**^
None	50 (13.2)	8 (4.75–12)	250.30		9.5 (2.75–12)	240.74	
Governmental	294 (77.8)	5 (2–8)	183.59		5 (0–9)	188.44	
Private	34 (9.0)	4 (2–5.25)	151.19		0 (0–3.25)	123.35	
*Place of Birth*				0.859^**b**^			0.604^**b**^
In Palestine	350 (92.6)	5 (2–8)	189.78		5 (0–10)	190.31	
Outside Palestine	28 (7.4)	6 (2–7)	185.98		2.5 (0–10)	179.36	
*Type of DM*				**0.009**^**b**^			** < 0.001**^**b**^
Type 1 DM	73 (19.3)	4 (2–6)	159.60		0 (0–6)	134.01	
Type 2 DM	305 (80.7)	6 (2–8.75)	196.66		6 (0–10)	202.78	
*Duration of DM (years)*				0.069^**a**^			0.271^**a**^
1–3	42 (11.1)	3.5 (2.0–7.0)	153.65		3.5 (0.0–11.0)	192.70	
4–5	42 (11.1)	4.0 (2.8–8.3)	186.48		3.0 (0.0–8.3)	164.24	
> 5	294 (77.8)	6.0 (2.0–9.0)	195.05		5.0 (0.0–9.3)	192.65	
*Treatment type*				**0.000**^**a**^			**0.009**^**a**^
Lifestyle modification	14 (3.7)	2.5 (0–4)	106.21		1 (0–14)	167.07	
Monotherapy	214 (56.6)	5 (2–8)	180.12		4 (0–9)	176.43	
Combined therapy	150 (39.7)	6 (3–10)	210.65		7 (1–11)	210.23	
*Insulin use*				**0.043**^**b**^			0.649^**b**^
Yes	201 (53.2)	6 (3–9.75)	200.14		4 (0–9)	187.13	
No	177 (46.8)	4 (2–8)	177.42		6 (0–10)	192.19	
*Co-morbidities*				** < 0.001**^**b**^			** < 0.001**^**b**^
Yes	247 (65.3)	6 (3–10)	210.20		7 (1–10)	209.46	
No	131 (34.7)	4 (2–6)	150.47		1 (0–8)	151.87	
*Total number of co-morbidities*				** < 0.001**^**a**^			** < 0.001**^**a**^
0	131 (34.7)	4.0 (2.0–6.0)	150.47		1.0 (0.0–8.0)	151.87	
1	194 (51.3)	6.0 (3.0–10.0)	212.51		7.0 (1.0–10.0)	203.38	
≥ 2	53 (14.0)	6.0 (2.0–10.5)	201.76		8.0 (2.5–13.5)	231.72	
*Regular Exercise*				** < 0.001**^**b**^			** < 0.001**^**b**^
Yes	108 (28.6)	3 (0–6)	146.69		0 (0–4)	127.50	
No	270 (71.4)	6 (3–9)	206.62		7 (1–11)	214.30	
*Alcohol intake*				**0.002**^**b**^			**0.041**^**b**^
Yes	11 (2.9)	0 (0–3)	90.95		0 (0–9)	124.18	
No	367 (97.1)	6 (2–8)	192.45		5 (0–10)	191.46	

UDI-6 Urinary Distress Inventory—Short Form, IIQ-7 Incontinence Impact Questionnaire—Short Form, BMI body mass index, NIS New Israeli Shekel (1 NIS = 0.29 US Dollars)

*Significant p-values are in bold

^a^Calculated by using the Kruskal–Wallis test

^b^Calculated by using the Mann–Whitney U test

### Clinical characteristics

Most of our participants had T2DM with a median duration of 14 years. Among all subjects, only 3.7% were on lifestyle modification, 56.6% were on a single therapy, and the rest (39.7%) used combination therapy. In addition, most subjects had different co-morbidities other than diabetes with a percentage of 65.3% (Table 1).

### Participants’ responses to the UDI-6 and IIQ-7 questions

Regarding urinary distress observations, subjects scored a median of 5.50 out of 18 points (Q1–Q3, 2.00–8.00) on the UDI-6 scale, and a median of 5 out of 21 points (Q1–Q3, 0.00–10.00) points for the IIQ-7 scale.

Tables 2 and 3 represent the distribution of responses to each question in urogenital symptom scales in diabetic patients. For example, 49.7% of the study population mentioned that their urinary symptoms affect their ability to do household chores. In addition, 51.3%, 48.1%, and 50% had limitations in recreational, entertaining and social activities (respectively). The scale also showed that 55.4% of the participants decreased their ability to travel more than 30 min by car or bus. Regarding the effect on emotional health, 54.2% mentioned some kind of nervousness and depression related to their condition and 52.1% described a frustrating feeling about their symptoms (Tables 2 and 3).

**Table 2:** Distribution of responses to each question in the urinary distress inventory short-form (UDI-6) on a four-point Likert scale ranged from 0 to 3 (not at all, a little bit, moderately, and greatly)

Do you experience and, if so, how much are you bothered by	Not at all n (%)	A little bit n (%)	Moderately n (%)	Greatly n (%)
“Frequent Urination?”	70 (18.5)	86 (22.8)	144 (38.1)	78 (20.6)
“Urine leakage related to urgency?”	166 (43.9)	120 (31.7)	69 (18.3)	23 (6.1)
“Urine leakage related to physical activity?”	209 (55.3)	106 (28.0)	46 (12.2)	17 (4.5)
“Small amounts of urine leakage?”	197 (52.1)	116 (30.7)	54 (14.3)	11 (2.9)
“Difficulty emptying your bladder or difficulty urinating?”	187 (49.5)	119 (31.5)	54 (14.3)	18 (4.8)
“Pain or discomfort in your lower abdominal, pelvic, or genital area?”	128 (33.9)	143 (37.8)	72 (19.0)	35 (9.3)

^a^Adapted from Uebersax et al. [28]

**Table 3:** Distribution of responses to each question in the incontinence impact questionnaire, short-form (IIQ-7) on a four-point Likert scale ranged from 0 to 3 (not at all, slightly, moderately, and greatly**)**

Has urine leakage (incontinence) affected your	Not at All n (%)	Slightly n (%)	Moderately n (%)	Greatly n (%)
“Ability to do household chores”	190 (50.3)	108 (28.6)	70 (18.5)	10 (2.6)
“Physical recreation such as walking, swimming or other exercises?”	184 (48.7)	118 (31.2)	58 (15.3)	18 (4.8)
“Entertaining activities (movies, concerts, etc.)?”	196 (51.9)	103 (27.2)	71 (18.8)	8 (2.1)
“Ability to travel by car or bus more than 30 min from home?”	168 (44.4)	101 (26.7)	89 (23.5)	20 (5.3)
“Participation in social activities outside your home?”	189 (50.0)	102 (27.0)	75 (19.8)	12 (3.2)
“Emotional health (nervousness, depression, etc.)?”	173 (45.8)	108 (28.6)	75 (19.8)	22 (5.8)
“Feeling frustrated?”	181 (47.9)	112 (29.6)	61 (16.1)	24 (6.3)

^a^Adapted from Uebersax et al. [28]

### Correlation between UDI-6, IIQ-7, and HbA1c readings

The responses on UDI-6 significantly correlated with their responses on the IIQ-7 scale and their last HbA1c readings (r = 0.546, p < 0.001 and r = 0.163, p-value < 0.001, respectively). Likewise, their responses to IIQ-7 also significantly correlated with their last HbA1c readings (r = 0.252, p < 0.001).

### Results of a univariate analysis

The correlations between each variable with the UDI-6 and IIQ-7 scores are presented in Table 1. Our analysis showed that female participants, those who are single, unemployed, have low levels of education or reside outside the city, had significantly higher scores on both scales. Similarly, both scores increase significantly with age and BMI. Additionally, certain clinical variables were significantly associated with UDI and IIQ-7 scores, such as type of diabetes and number of co-morbidities. However, there was no statistically significant association between the duration of DM and the scores of both scales. On the other hand, using insulin as part of the treatment regimen was associated with an increased UDI-6 score but without a similar increase in the IIQ-7 score.

Regarding urinary distress observations, subjects scored a median of 5.50 out of 18 points (Q1–Q3, 2.00–8.00) on the UDI-6 scale, and a median of 5 out of 21 points (Q1–Q3, 0.00–10.00) points for the IIQ-7 scale.

Participants’ responses on UDI-6 were significantly correlated with their responses on the IIQ-7 scale (r = 0.546, *p* < 0.001). Likewise, their responses to UDI-6 and IIQ-7 were significantly correlated with their last HbA1c readings (0.163, *p* < 0.001; r = 0.252, *p* < 0.001, respectively).

### Results of multiple linear regression analysis

All variables with a significant P-value in univariate analysis were entered in the multiple linear regression analysis. Consequently, this analysis was constructed according to BMI, residency, age, sex, marital status, education level, job, income, type of insurance, type of DM, type of treatment, insulin use, co-morbidities, total number of co-morbidities, regular exercise, alcohol intake, and last HbA1c reading. Multiple linear regression models that were estimated to explore associations with the UDI-6 score found that marital state, residency, type of insurance, regular exercise, and alcohol intake were significantly and negatively correlated with the UDI-6 score (*p*-values: 0.006, 0.038, < 0.001, 0.001, 0.042, respectively). However, sex, insulin use and the presence of co-morbidities were significantly and positively correlated with the UDI-6 score (*p*-values: 0.042, 0.009, and 0.007, respectively). According to the regression models of the IIQ-7 score, marital state, type of insurance, and regular exercise were significantly and negatively correlated with IIQ-7 (*p*-values: 0.039, < 0.001, and < 0.001, respectively). However, age and BMI were significantly and positively correlated with the IIQ-7 score (*p* values: 0.040 and < 0.001, respectively). There was no evidence of multicollinearity between the independent variables (Tables 4 and 5).

**Table 4:** Multiple linear regression analysis of UDI-6 score

Model	Unstandardized coefficients		Standardized coefficients	p-value*	Collinearity statistics
	B	SE	Beta		VIF
(Constant)	2.370	1.947		0.224	
Age	0.056	0.256	0.018	0.826	3.207
Sex	0.968	0.474	0.116	**0.042**	1.557
BMI	0.305	0.361	0.051	0.400	1.785
Marital status	− 1.249	0.453	− 0.146	**0.006**	1.346
Residency	− 0.649	0.312	− 0.101	**0.038**	1.132
Education level	0.311	0.461	0.037	0.500	1.436
Job	0.026	0.554	0.003	0.963	1.921
Income	0.498	0.284	0.097	0.080	1.459
Type of Insurance	− 1.962	0.432	− 0.222	** < 0.001**	1.142
Type of DM	1.438	1.001	0.137	0.152	4.335
Type of treatment	0.011	0.464	0.001	0.981	1.819
Insulin use	1.479	0.561	0.178	**0.009**	2.175
co-morbidities	2.240	0.829	0.257	**0.007**	4.325
Total number of co-morbidities	− 0.655	0.594	− 0.105	0.271	4.355
Regular exercise	− 1.494	0.456	− − 0.162	**0.001**	1.179
Alcohol intake	− 2.467	1.209	− 0.100	**0.042**	1.147
Last HbA1c reading	0.043	0.139	0.016	0.757	1.222

**Table 5:** Multiple linear regression analysis of IIQ-7 score

Model	Unstandardized coefficients		Standardized coefficients	p-value*	Collinearity statistics
	B	SE	Beta		VIF
(Constant)	1.002	2.173		0.645	
Age	0.617	0.299	0.159	**0.040**	3.130
Sex	0.117	0.553	0.011	0.832	1.509
BMI	1.515	0.428	0.206	** < 0.001**	1.783
Marital status	− 1.105	0.533	− 0.104	**0.039**	1.327
Residency	− 0.182	0.366	− 0.023	0.619	1.106
Education level	0.255	0.545	0.024	0.640	1.429
Job	− 1.164	0.654	− 0.107	0.076	1.905
Income	− 0.064	0.336	− 0.010	0.848	1.448
Type of Insurance	− 1.800	0.512	− 0.164	** < 0.001**	1.141
Type of DM	− 0.200	1.014	− 0.015	0.844	3.167
Type of treatment	− 0.195	0.455	− 0.021	0.668	1.248
co-morbidities	0.194	0.982	0.018	0.844	4.317
Total number of co-morbidities	0.548	0.704	0.071	0.437	4.353
Regular exercise	− 2.619	0.538	− 0.229	** < 0.001**	1.169
Alcohol intake	− 0.064	1.421	− 0.002	0.964	1.128
Last HbA1c reading	0.287	0.163	0.084	0.079	1.195

## Discussion

This study shows that LUTS is a prevalent problem among DM patients with variable presentations. The LUTS has been studied frequently in Palestine among different cohorts but not among DM patients [7, 8, 39]. DM patients represent a special category as LUTS may be attributed to several reasons, such as UTIs, bladder dysfunction, and bladder outlet obstruction [40, 41]. However, in this study, we screened for LUTS, which had no obvious clinically visible reason, such as UTIs or neurological causes. The median UDI-6 score in our study was 5.50, whereas the median of IIQ7 was 5.00. Both scales showed significant correlations with each other. Therefore, the study confirms the direct negative impact of LUTS on diabetic patients’ life. This new concept in Palestine shows a compelling need that strategies should be taken at all healthcare levels to identify such problems at an early stage so avoid physical and mental exhaustion among DM patients.

The results of our study come parallel with international results as high BMI, lack of exercise and co-morbidities increase LUTS severity [33] [42]. Nevertheless, high BMI and lack of physical activity are associated with LUTS regardless of the status of DM [43]. Furthermore, it is evident that weight loss and regular exercise improve LUTS and treat urinary incontinence among females [44]. Therefore, weight reduction and increased physical activity in a diabetic not only improve DM control and prevent serious complications but also helps DM patients to improve LUTS [45, 46].

Surprisingly, the level of HBA1c and the duration of diabetes are not shown to affect LUTS in our cohort of patients. However, several studies show a positive correlation between the degree of HBA1c and stress and urge urinary incontinence [47, 48]. The high level of HBA1c reflects poorly controlled DM, which is attributed to DM-related complications and end-organ damage, including bladder cystopathy [14, 41, 49]. Thus, we still believe that poorly controlled DM and high HBA1c are related to LUTS, especially since our results show that DM patients taking a combination of medication and insulin are more likely to have LUTS [49].

Females had higher scores on both scales, which indicates that the severity of LUTS is more common among female DM patients. This is compatible with other international studies that showed LUTS are common in women and cause great distress and embarrassment [50–52]. However, it is mandatory to assess for UTI among DM females with LUTS as LUTS, mainly incontinence exacerbates during active UTI, which is a treatable common problem among DM females [53, 54]. However, screening for asymptomatic bacteriuria is not routinely required among DM female patients, and treating asymptomatic bacteriuria does not change LUTS's rate or severity [54]. On the other hand, LUTS among diabetic males creates a challenge to diagnose and treat for several reasons, such as concomitant Benign Prostatic Enlargement in elderly diabetic patients, which may complicate the diagnostic algorithm. Thus, the pressure-flow study is a wise step for elderly diabetic patients to accurately diagnose the underlying problem, especially before potential surgical treatment such as TURP [6].

Taking into consideration all the above facts, LUTS does affect diabetic patients. Several factors may play a role in developing or worsening LUTS. Early identification of LUTS helps DM patients to avoid struggling with such annoying symptoms. Female DM patients should be offered a questionnaire to screen for LUTS. If LUTS is confirmed, appropriate evaluation and management should be followed. DM patients can take several actions to decrease LUTS, such as regular exercise, weight loss, and tight control of DM.

## Limitations and strengths

This was the first study in Palestine to examine the prevalence and severity of symptoms of urinary distress in diabetic patients. However, one of the limitations of this study was the cross-sectional design, which prevented us from interpreting the causality of the significant associations in our results. Another limitation is that it took place in only two clinics, which may limit the generalizability of our data to all diabetic patients in Palestine.

## Conclusions

LUTS is significant morbidity among DM patients. Several factors, such as age, BMI, and co-morbidities, exacerbate LUTS, which can be modified and controlled. Thus, a screening questionnaire should be offered mainly to DM female patients to address the severity of LUTS and its impact on QoL.

## Data Availability

Due to privacy, the datasets used and/or analysed during the current study are available from the corresponding author on reasonable request.

## Abbreviations

LUTS: Lower urinary tract symptoms
DM: Diabetis Mellitus
QoL: Quality of life
UDI-6: Urogenital Distress Inventory-6
IIQ-7: Incontinence Impact Questionnaire-7
BMI: Body mass index
UTIs: Urinary tract infections
Hba1c: Hemoglobin A1C
kg: Kilograms
m2: Meters squared
IBM-SPSS: Statistical Package for the Social Sciences
